# Clinical and histopathological features resembling those of human focal segmental glomerulosclerosis in a cat with nonimmune-mediated glomerulonephropathy

**DOI:** 10.1186/s12917-015-0569-4

**Published:** 2015-10-06

**Authors:** Go Sugahara, Satoshi Hosaka, Takayuki Mineshige, Junichi Kamiie, Kinji Shirota

**Affiliations:** Laboratory of Veterinary Pathology, School of Veterinary Medicine, Azabu University, 1-17-71 Fuchinobe, Chuo-ku, Sagamihara, Kanagawa 252-5201 Japan; Hosaka Animal Hospital, Sagamihara, Kanagawa Japan

**Keywords:** Cat, Renal biopsy, Glomerulonephropathy, Focal segmental glomerulosclerosis

## Abstract

**Background:**

Nonimmune-mediated glomerulonephropathies are rarely reported in domestic animals with the exception of amyloidosis. Here we describe the pathological features and clinical course of a feline with protein-losing nonimmune-mediated glomerulonephropathy characterized by segmental glomerulosclerosis and severe podocyte injury.

**Case presentation:**

A castrated male Japanese domestic cat aged 3 years and 8 months had hypertension, persistent proteinuria, and azotemia. Microscopic examination of a renal biopsy revealed many glomeruli with adhesion to the Bowman’s capsule and segmental sclerosis. The most characteristic ultrastructural glomerular feature was severe podocyte foot process effacement. No electron-dense deposits were observed. Immunofluorescence revealed no immune deposits, but abnormal expression of nephrin and podocin was detected in the glomeruli. These findings resemble those of human focal segmental glomerulosclerosis. The cat temporarily responded to treatment with angiotensin-converting enzyme inhibitors and prednisolone administration but died of progressive renal failure 32 months after biopsy.

**Conclusions:**

The cat was diagnosed with nonimmune mediated glomerulonephropathy because of the absence of immune deposits and severe podocyte injury. To our knowledge, this is the first report of nonimmune-mediated glomerulonephropathy in a cat resembling human focal segmental glomerulosclerosis.

## Background

Immune-mediated glomerulonephritis has been reported in dogs and cats as a common renal disease that causes chronic renal failure [1]; however, with the exception of amyloidosis, nonimmune-mediated glomerulonephropathies are rarely reported in domestic animals. Here we describe the pathological features and clinical course of a cat with protein-losing nonimmune-mediated glomerulonephropathy characterized by segmental glomerulosclerosis and severe podocyte injury.

## Case presentation

**Fig. 1:**
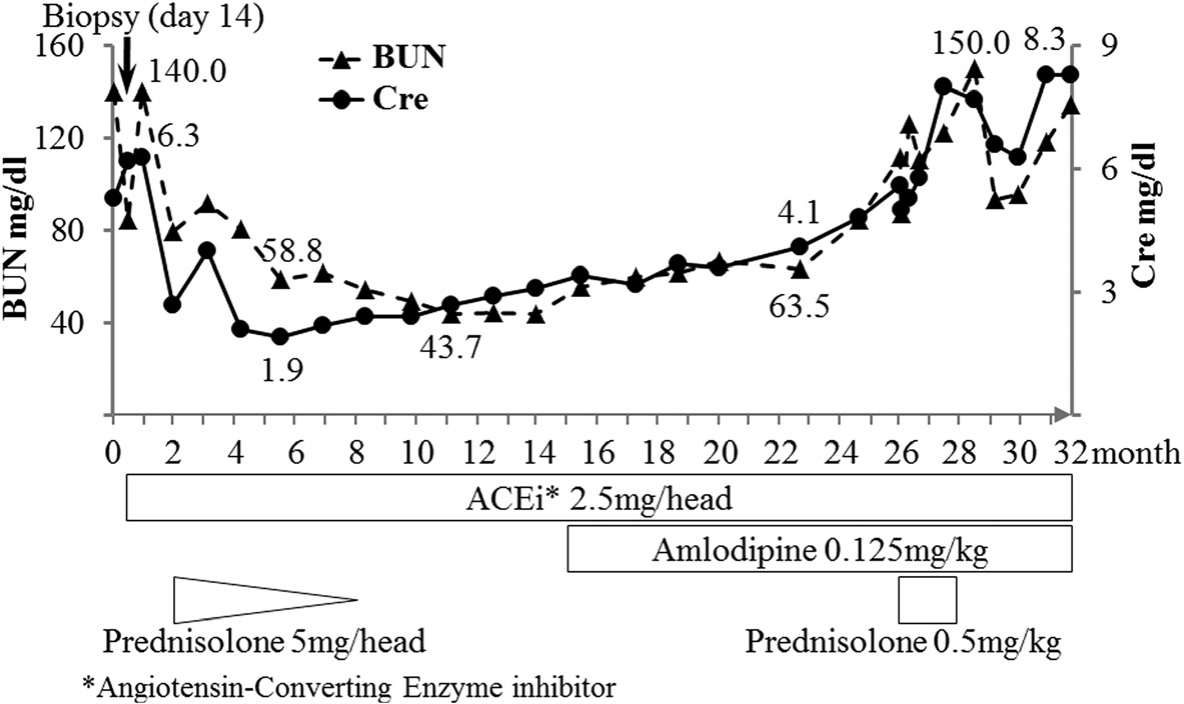
Treatment and changes in the levels of blood urea nitrogen (BUN) and serum creatinine (Cre) during the disease course

This Japanese domestic cat was a castrated male aged 3 years and 8 months with anorexia, weight loss, and polyuria/polydipsia for a few months. Clinical examinations before treatment and renal biopsy revealed a heart murmur, hypertension (systolic blood pressure of 220 and 160 mmHg at 10 and 5 days before biopsy, respectively), renal enlargement, and abnormalities of blood biochemistry and urinalysis, such as azotemia (Fig. 1) and persistent proteinuria (a urinary protein-to-creatinine ratio of 2.19 and 300–1000 mg/dL protein level using reagent strips).

Two-third of the renal biopsy tissue obtained from the cat was fixed in 10 % neutral-buffered formalin and embedded in paraffin wax. After processing, sections (3 μm) were stained with hematoxylin and eosin (HE) and periodic acid-Schiff (PAS) for histological examination. For immunofluorescence (IF) using goat anti-nephrin (Santa Cruz Biotechnology, Santa Cruz, CA) or rabbit anti-podocin (Sigma-Aldrich, St Louis, MO, USA) antibodies, dewaxed sections were pretreated with Target Retrieval Solution, pH 9.0 (Nichirei Corp., Tokyo, Japan) at 121 °C for 5 min in an autoclave for nephrin or with trypsin (Sigma; T7168) at 37 °C for 30 min for podocin. The secondary antibodies were Alexa Fluor 488-conjugated rabbit anti-goat immunoglobulin (Ig)G (Invitrogen, Tokyo, Japan) or Alexa Fluor 488-conjugated goat anti-rabbit IgG (Invitrogen). Renal tissue from a 10-year-old female mixed breed cat with mild and focal interstitial nephritis was used for comparison.

A small piece of fresh biopsy tissue was embedded in an optimal cutting temperature compound (Sakura Finetek, Tokyo, Japan). Cryosections and paraffin sections were prepared to detect immune deposits. Direct IF studies using fluorescein isothiocyanate (FITC)-conjugated anti-cat IgG (Kirkegaard & Perry Laboratories, Inc., Gaithersburg, MD, USA) and indirect immunostaining using anti-cat C3 primary antibodies (Biogenesis, Poole, UK) and FITC-conjugated secondary antibodies (Chemicon, Temecula, California) were performed. An FSX100 fluorescence microscope (OLYMPUS, Tokyo, Japan) was used for examination.

For ultrastructural examination, another small piece of the biopsy tissue was fixed in 2.5 % glutaraldehyde and postfixed in 1 % OsO_4_. Fixed specimens were then dehydrated using ascending grades of alcohol and embedded in epoxy resin. Ultrathin sections were stained with uranyl acetate and lead citrate and observed using a JEOL 1210 transmission electron microscope (JOEL, Tokyo, Japan) at 80 k.

The treatment and levels of blood urea nitrogen (BUN) and serum creatinine (Cre) during the disease course are shown in Fig. 1. After renal biopsy, the administration of a sustained course of angiotensin-converting enzyme (ACE) inhibitor (benazepril, Novartis Pharma K.K., Tokyo, Japan) and a tapering course of prednisolone were immediately started. For approximately 1 year, the levels of BUN and Cre were decreased. However, approximately 15 months later, both levels gradually began to increase. Amlodipine and prednisolone were administered without any noticeable effect. The cat died from the progression of renal failure with anorexia and anemia approximately 32 months after renal biopsy. During the disease course, persistent proteinuria (30–100 mg/dl using reagent strips) was observed despite treatment. Necropsy was not allowed by the owner.

**Fig. 2:**
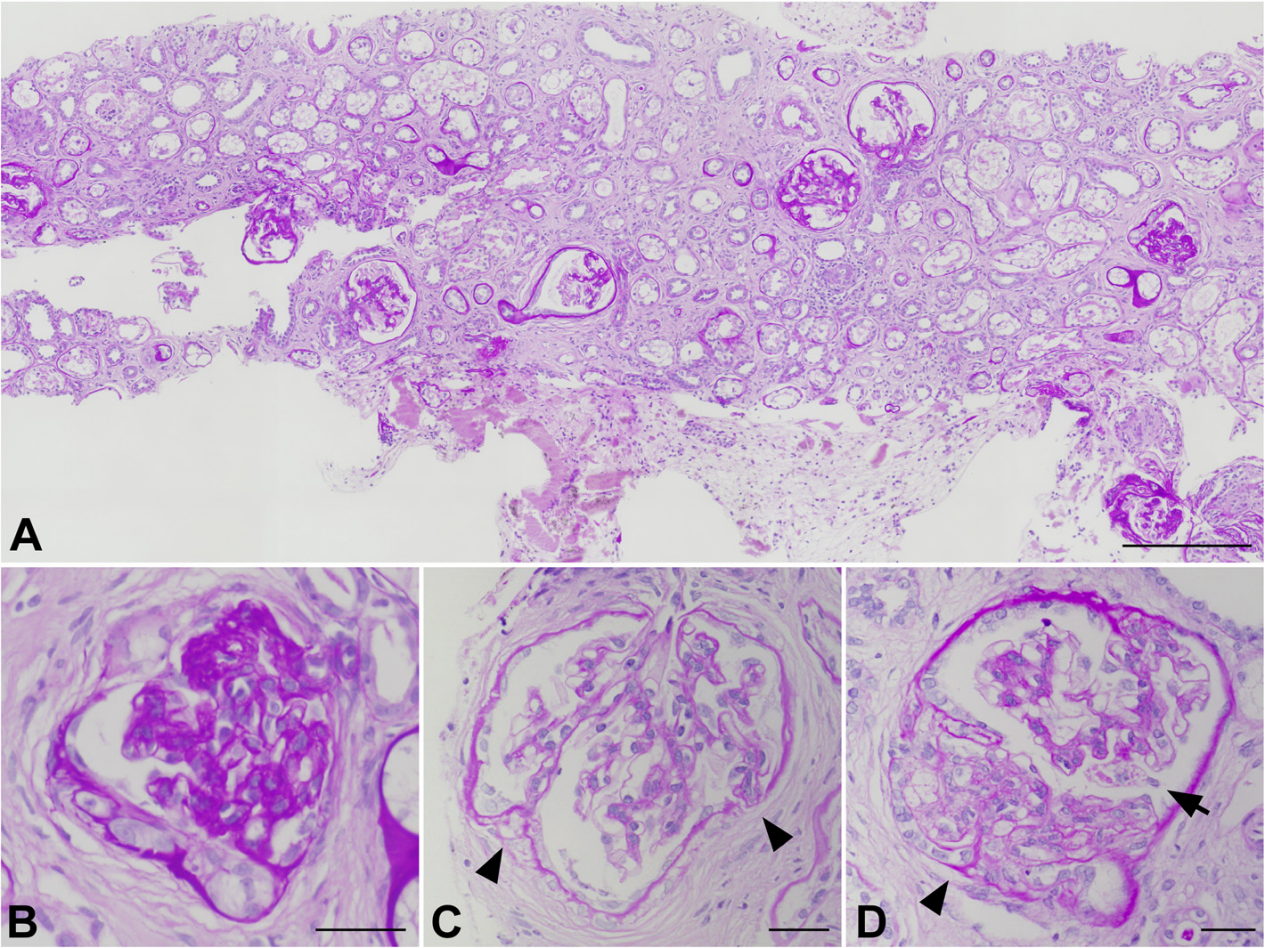
Light microscopic features of the biopsy specimen: (**a**) low magnification and (**b** to **d**) high magnification images of the glomeruli. Infiltration of lymphocytes and plasma cells in the interstitium, severe interstitial fibrosis and tubular atrophy were observed (**a**). The glomeruli show global sclerosis (**b**), multiple adhesion to Bowman’s capsule (**c**: arrowheads), segmental sclerosis with increased matrix and adhesion (**d**: arrowheads) and occasional hyaline droplets in podocyte cell bodies (**d**: arrow). Periodic acid-Schiff stain. Scale bars: 30 μm

Microscopically, the biopsy specimen contained 12 glomeruli with the following characteristics: one showed global sclerosis (8.3 %), four (33.3 %) showed adhesion to the Bowman’s capsule, and four (33.3 %) showed segmental sclerosis with increased matrix (Fig. 2). The regions without sclerosis or adhesion in these affected glomeruli and the remaining three (25 %) glomeruli appeared normal, except for occasional hyaline droplets in the podocytes. Diffuse mononuclear-cell infiltration and fibrosis were observed in the interstitium.

**Fig. 3:**
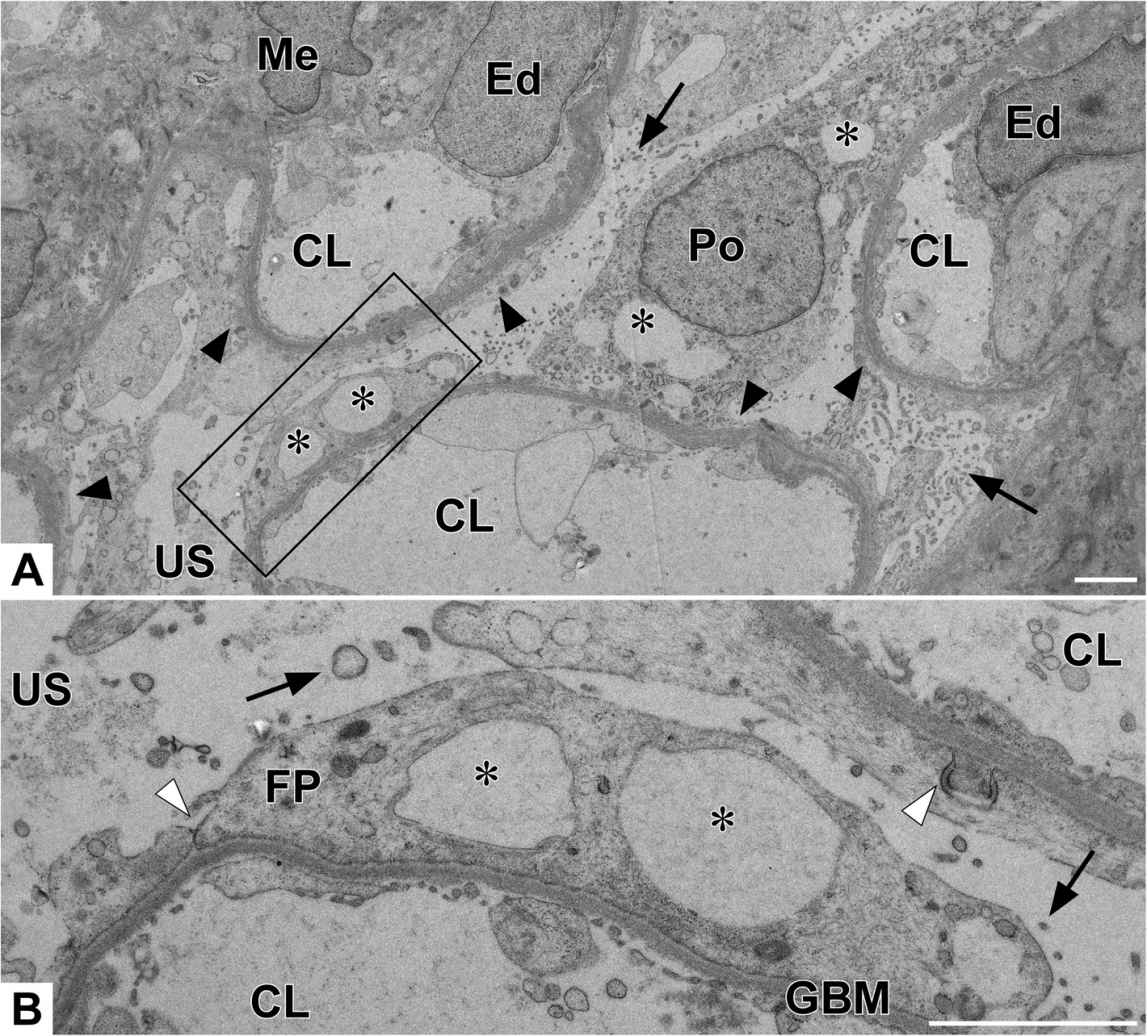
Transmission electron microscopic features of the biopsy specimen: (**a**) low and (**b**) high magnifications of a region in the frame in (**a**). Severe and global foot process effacement (arrowheads), vacuolization (asterisks), formation of microvilli (arrows), and tight junctions between foot processes (white arrowheads) are observed. CL, capillary lumen; Ed, endothelial cell; FP, foot process; GBM, glomerular basement membrane; Me, mesangial cell; Po, podocyte; US, urinary space. TEM, transmission electron microscopy. Scale bars: 2 μm

Examination using transmission electron microscopy revealed a distinct lesion in the glomeruli characterized by severe and global foot process (FP) effacement without electron-dense deposits, and there were no obvious glomerular basement membrane (GBM) abnormalities or cellular infiltration (Fig. 3) The podocytes had global changes such as cytoplasmic vacuolation, the formation of tight junctions between FPs, and microvilli formation on the free surface.

**Fig. 4:**
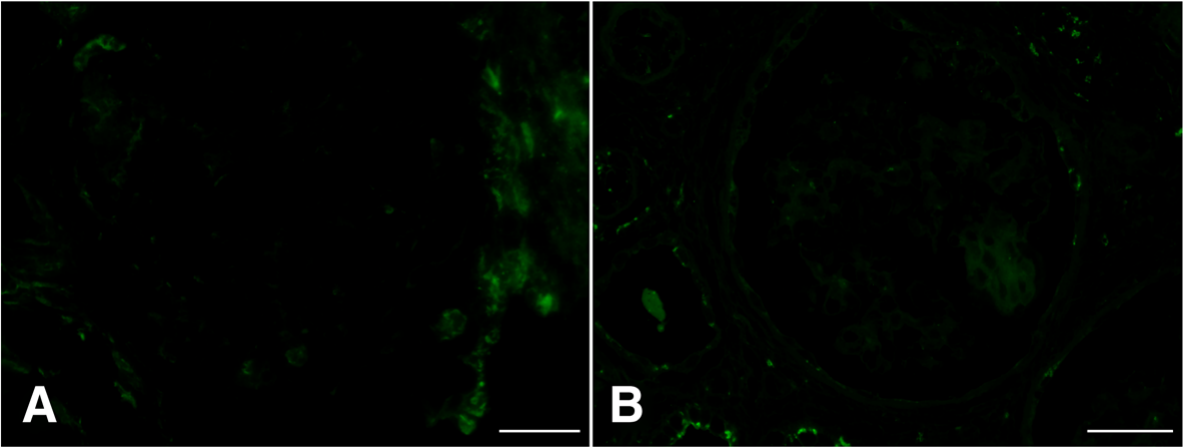
Immunofluorescence analysis of IgG and C3 expression in the glomeruli. No signal for feline IgG (**a**) and C3 (**b**) was observed in the glomeruli. Scale bars: 30 μm

**Fig. 5:**
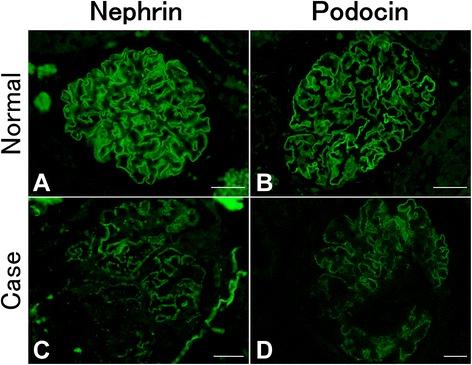
Immunofluorescence analysis of nephrin and podocin expression in the glomeruli. Expression of nephrin and podocin (**c** and **d**) was decreased, and staining exhibited a coarse granular pattern compared with the sharp linear pattern of normal glomeruli (**a** and **b**). Scale bars: 30 μm

Immunohistochemical analysis did not detect the deposition of IgG and C3 in the glomeruli (Fig. 4). In the normal cat glomeruli, the labeling of nephrin and podocin exhibited a sharp, linear pattern along the GBM, as previously reported. In contrast, in the present case, the expression of those proteins was decreased, and the labeling pattern was granular (Fig. 5).

## Discussion

We regarded this renal biopsy case as a primary glomerulonephropathy with tubulointerstitial changes. Although the light microscopic findings were similar to those of chronic interstitial nephritis (CIN), which is the most common renal disease of cats [2–4], feline CIN commonly develops in animals older than this one and is not accompanied by persistent proteinuria. Podocyte injury is closely related to proteinuria, and persistent proteinuria causes tubulointerstitial injury as a common mechanism of proteinuric nephropathy in humans and animals [3–5]. Nephrin and podocin are expressed in FPs and slit diaphragms and play a critical role in selective filtration. Altered expression of these proteins indicates podocyte injury related to proteinuria in humans and in animal models [6–8]. Therefore, in addition to ultrastructural changes, decreased expression and the altered expression pattern of nephrin and podocin indicate that podocyte injury and dysfunction likely occurred as a primary event in the present case.

The histopathological, IF, and electron microscopic findings suggested nonimmune-mediated pathogenesis in the present case. Nonimmune-mediated glomerulonephropathy related to podocyte injury in cats has been rarely reported, and to our knowledge, there is only one report of minimal-change glomerulopathy in a cat [9] in which the renal glomeruli show severe FP effacement without glomerulosclerosis. In the present study, the glomeruli showed irreversible lesions such as adhesion and sclerosis as well as severe podocyte injury, and these morphological changes resemble those of human focal segmental glomerulosclerosis (FSGS). FSGS is a major cause of nephrotic syndrome and subsequent progression to end-stage renal disease in humans [10, 11], which was also reported in a dog [12].

The diagnosis of FSGS is based on the following light microscopic findings: glomerular sclerosis and tuft collapse are focal (some glomeruli, but not all) and segmental (a segment of the glomerulus is affected). However, in practice, diffuse or global glomerulosclerosis is sometimes observed in human and canine FSGS and its animal models, depending on the disease stage [8, 12, 13]. Therefore, the renal disease in the present case may be more advanced stage of FSGS, because of high frequency of affected glomeruli and tubulointerstitial lesions in association with renal failure.

The key pathogenic mechanism of FSGS is the progressive damage of podocytes and their detachment from the GBM followed by capillary tuft adhesion to the Bowman’s capsule and sclerosis [10, 11]. FSGS encompasses a number of clinicopathologic syndromes sharing this common glomerular lesion, including primary (or idiopathic) and secondary FSGS (related to a variety of causes). Hypertension is an important underlying comorbidity in humans with secondary FSGS [13]. However, hypertension in the present case might have been caused by chronic renal failure, and its relationship to the pathogenesis of FSGS is unclear because of the lack of long-term monitoring of blood pressure.

FSGS in humans is less responsive to steroid therapy [11, 14]. Here the cat temporarily responded to an ACE inhibitor and steroid therapy but died due to the progression of renal failure. However, as previously mentioned, renal function was diminished at the time of renal biopsy, and we were therefore unable to evaluate the exact therapeutic effect.

## Conclusions

This case report demonstrates the importance of examination of renal biopsies using IF and electron microscopy as well as light microscopy. Moreover, to our knowledge, this is the first report of nonimmune-mediated glomerulonephropathy in a cat that resembles human FSGS.

## Abbreviations

HE: Hematoxylin and eosin
PAS: Periodic acid-Schiff
IF: Immunofluorescence
BUN: Blood urea nitrogen
Cre: Creatinine
ACE: Angiotensin-converting enzyme
FP: Foot process
GBM: Glomerular basement membrane
CIN: Chronic interstitial nephritis
FSGS: Focal segmental glomerulosclerosis
